# A Numerical and Experimental Study of Adhesively-Bonded Polyethylene Pipelines

**DOI:** 10.3390/polym11091531

**Published:** 2019-09-19

**Authors:** Antoine Guilpin, Geoffrey Franciere, Lewis Barton, Matthew Blacklock, Martin Birkett

**Affiliations:** 1 ENSIAME, Université de Valenciennes et du Hainaut-Cambrésis, Le Mont-Houy, 59313 Valenciennes CEDEX 9, France; aguilpin14@gmail.com (A.G.); gfranciere@gmail.com (G.F.); 2 Rosen Group, Quorum Business Park, Newcastle upon Tyne NE12 8BS, UK; lbarton@rosen-group.com; 3 Department of Mechanical and Construction Engineering, Northumbria University, Newcastle upon Tyne NE1 8ST, UK; matthew.blacklock@northumbria.ac.uk

**Keywords:** polyethylene, adhesively-bonded joints, double cantilever beam, end-notched flexure, finite element analysis, cohesive zone model

## Abstract

Adhesive bonding of polyethylene gas pipelines is receiving increasing attention as a replacement for traditional electrofusion welding due to its potential to produce rapid and low-cost joints with structural integrity and pressure tight sealing. In this paper a mode-dependent cohesive zone model for the simulation of adhesively bonded medium density polyethylene (MDPE) pipeline joints is directly determined by following three consecutive steps. Firstly, the bulk stress-strain response of the MDPE adherend was obtained via tensile testing to provide a multi-linear numerical approximation to simulate the plastic deformation of the material. Secondly, the mechanical responses of double cantilever beam and end-notched flexure test specimens were utilised for the direct extraction of the energy release rate and cohesive strength of the adhesive in failure mode I and II. Finally, these material properties were used as inputs to develop a finite element model using a cohesive zone model with triangular shape traction separation law. The developed model was successfully validated against experimental tensile lap-shear test results and was able to accurately predict the strength of adhesively-bonded MPDE pipeline joints with a maximum variation of <3%.

## 1. Introduction

Due to its unique combination of properties, polyethylene (PE) is now the material of choice for low-pressure water and gas pipeline systems [1]. It offers numerous advantages over traditional metal pipelines such as lower cost, higher strength-to-weight ratio, increased flexibility, and superior corrosion resistance and chemical inertness [2,3,4,5,6]. Perhaps the most attractive feature of PE pipeline systems is the ability to rapidly fuse sections together to form joints with strength equivalent to the parent material. This allows networks with a minimum design life of 50 years [7]. Fusion can be achieved using hot iron and electrofusion techniques to produce a range of joint geometries, such as butt welds, socket joints and saddle joints, as well as applying repair patches. Although, in theory, these techniques should offer a reliable joining solution, fusion welding is a complex process requiring skilled operatives and its success is highly reliant upon multiple process parameters such as fusion pressure, melt temperature, heat soak and dwell times, and pipe cleanliness, ovality and alignment [7]. Any defects in the fusion zone can act as stress initiation sites for slow crack growth that will propagate through the fused joint, eventually leading to failure [3,8]. Along with third party damage, poor quality fusion joints are reported to be the main threat to PE pipeline integrity. According to the Plastic Pipe Database Committee’s latest 2018 status report, 78.5% of PE pipeline failures or leaks were due to problems with joints (16.1%) and fittings (62.4%), with the remaining 21.5% being attributed to faults in the pipeline itself (15%) or not being recorded (6.5%) [9]. Furthermore, producing high quality fusion joints can be a time consuming process that can become particularly disruptive and costly if the pipeline to be repaired is under a busy highway or walkway due to difficulties accessing the repair site with the electrofusion welding equipment.

One alternative method to fusion joining that is receiving increasing attention is adhesive bonding. It has the potential to reduce repair site access issues and produce rapid and low-cost joints with structural integrity and pressure tight sealing. Although adhesive bonding of PE has often been overlooked due to its low surface free energy, recent technological advancements in adhesive science and surface pre-treatments mean that it may now be a realistic alternative to fusion joining. Previous studies by the authors have highlighted this potential through successful bonding of PE using structural acrylic adhesives both at room [10] and low temperatures [11]. Pressure-tight joints with adequate mechanical strength were achieved for medium density polyethylene (MDPE) tapping tees bonded to MDPE gas pipelines. Although some encouraging experimental results have been reported thus far, there has been limited attention given to developing a theoretical understanding and a numerical model of the adhesively bonded assembly, which can be used to efficiently predict the strength of joints with modified geometries under various loading conditions.

Numerical modelling via the Finite Element Method (FEM) has been successfully used to analyse the strength of adhesive joints for almost 50 years [12]. Early models used the strength of materials and fracture mechanics methods to predict the strength of single lap joint assemblies [13,14]. Although these techniques can give reasonable results, analysis of strength using stress/strain criteria is highly mesh dependent due to stress singularities at the ends of the overlapping joint [15,16], while fracture analysis using linear elastic fracture mechanics (LEFM) requires an initial crack or inherent flaw in the joint to allow the calculation of stress or strain intensity [17].

A more recent approach, that has been developed to overcome these shortcomings and give a much more accurate strength prediction, is the cohesive zone model (CZM) [18,19]. The concept of the CZM was first proposed around 1960 to describe damage under static loads ahead of the crack tip [20,21], before first being used in FEM in 1976 to study crack growth in cementitious materials [22]. It was not until 1995 that a CZM was used by Crocombe et al. for the purpose of simulating adhesive joints [23]. The CZM can model both static and fatigue behaviour of adhesive joints at local and global levels. It combines the strength of materials and fracture based methods to diagnose the current state of damage along the adhesive joint, which results in progressive degradation of the material stiffness before failure [24]. Unlike classical fracture based approaches, CZMs have the ability to simulate damage initiation and growth without the need of an initial crack or flaw. The improved accuracy of the CZM over conventional LEFM is attributed to the ability to develop different shapes for the cohesive laws, depending on the properties of the joint interface being simulated. This relationship between stresses and relative displacements is defined as the traction separation law (TSL) and is usually of triangular or trapezoidal shape for typical structural materials. TSLs of predefined shape define the required cohesive parameters and require important properties of the bond interface such as the energy release rate and cohesive strength in both tension (failure mode I) and shear (failure mode II) [25].

These properties can be determined using three different techniques; the inverse method, the property identification method and the direct method [26]. The inverse method determines the cohesive parameters of a pre-defined TSL using a manual iterative process to tune the FEM load-displacement (*P–δ*) curve with experimental data [27], while the property identification method uses specific tests to identify each of the individual parameters [28]. These first two techniques require an initial estimation of the TSL shape based on the adhesive type, loading and environmental conditions and it can be difficult to perform the specific tests to obtain a correlation between the required properties using the property identification method [25,26]. Conversely, in the direct method, the precise shape of the TSL of a specific adhesive or interface is directly determined for failure in mode I and mode II using experimental data of double cantilever beam (DCB) and end-notched flexure (ENF) fracture tests. Crack tip opening displacements are recorded during these tests and used to calculate the respective strain energy release rates in each pure failure mode [19]. There are numerous examples of where CZMs utilizing DCB and ENF fracture tests and varying cohesive law shapes have been successfully used to numerically model the behaviour of adhesively-bonded joints. Carlberger and Stigh [29] studied the effect of adhesive layer thickness in the range 0.1 to 1.6 mm on the CZM shape and found it to vary from a rough triangular shape at low thicknesses to a trapezoidal shape at higher thicknesses. Campilho et al. [30] used numerical FEM incorporating a CZM with a trapezoidal shape in modes I and II to simulate a thin ductile adhesive layer in single strap repairs on laminated composites and found excellent agreement with experimental data. Kafkalidis and Thouless [31] also found that the CZM approach with a trapezoidal law gave excellent correlation with experimental results for symmetric and asymmetric single lap joints bonded with ductile adhesives, giving accurate predictions of failure loads, displacements and deformations in the joints. Campilho et al. [25] also recently studied the effect of the cohesive law shape to predict the strength of adhesively bonded single lap joints. They found that the trapezoidal shape provides the best fit with experimental data for ductile adhesives, while the influence of the CZM shape can be neglected when using brittle adhesives, and the more straightforward triangular shape law can be used to give results faster, on account of easier convergence, without compromising accuracy.

In this work, a FEM model was developed using a CZM with a triangular-shaped TSL to accurately predict the strength of MDPE gas pipeline material adhesively bonded in a single lap joint configuration using a methylmethacrylate (MMA)-based structural adhesive. The energy release rate and cohesive strength in failure mode I and II were determined directly using the DCB and ENF fracture tests and used to create the numerical model. The model was successfully validated against experimental tensile lap-shear test results to accurately predict the strength of the adhesively-bonded assembly.

## 2. Materials and Methods

All adherend substrates used in this investigation were cut from 250 mm diameter, 18 mm thick MDPE (PE80) yellow gas pipeline (GPS PE Pipe Systems, Huntingdon, UK). The sections of pipe were then machined using a VRX i-500 CNC machine (Mazak, Oguchi, Aichi Prefecture, Japan) to the required substrate dimensions for each physical test and then left to condition at room temperature (20 °C) and 40% relative humidity (RH) for 168 h. These substrates were bonded in the required configuration using a two-part MMA based structural adhesive (Easy-Mix PE-PP 45, Weicon, Munster, Germany). The key properties of the substrate and adhesive materials are presented in Table 1 [11].

Prior to assembly, the bond surfaces of the substrates were cleaned using Weicon solvent spray surface cleaner and wiped dry with a clean cloth. This process is performed to maximise the degree of intimate molecular contact to try to achieve a cohesive failure in the adhesive joint [26]. The bondline thickness for all samples was controlled to 0.2 mm using a combination of PTFE shims at the overlap edges and via 0.2 mm glass particles within the adhesive. Once assembled, all specimens were cured for 24 h at 20 °C and 40% RH and then tested immediately. All physical tests were carried out at 20 °C and 40% RH using a 3382 tensile testing machine (Instron, High Wycombe, UK) with a 100 kN load cell. A minimum sample of seven specimens were tested for each condition with at least four valid results always reported.

### 2.1. Material Characterisation

Important properties of the MDPE and acrylic adhesive materials were determined for input into the FEM model. The mechanical properties of the MDPE substrate were validated via tensile testing in accordance with ASTM D638-14 [32]. Specimens were cut from the MDPE pipeline and prepared in a flat dog bone shape, 246 mm long with a gauge length of 50 mm and cross-sectional gauge area of 15 × 10 mm. Specimens were mounted in the 3382 tensile testing machine with a distance between grips of 115 mm and strained under a crosshead speed of 2.0 mm/min.

**Figure 1:**
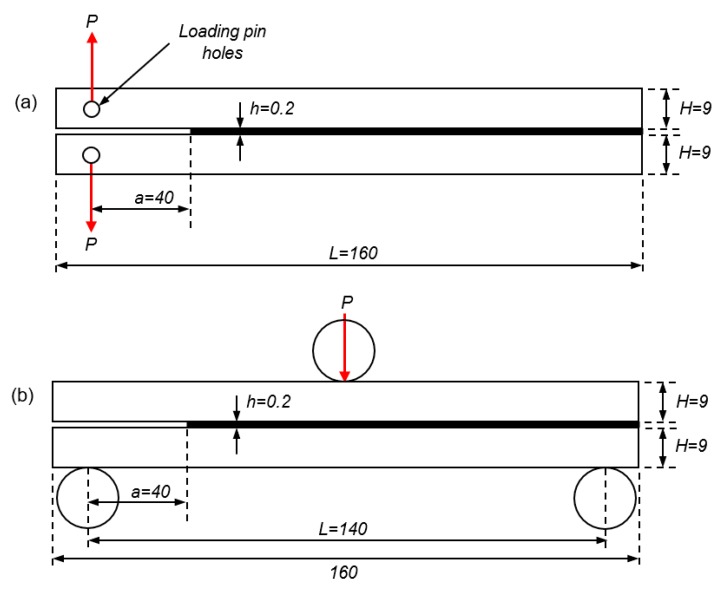
Specimen geometry for (**a**) the DCB test, and (**b**) the ENF test (dimensions in mm). Specimen breadth, *b* = 25 mm.

Mode I and II fracture toughness of the adhesive were determined by DCB and ENF tests, respectively. The DCB specimen dimensions and testing were in accordance with ASTM D3433-99 [33] and BS ISO 25217 [34], (Figure 1a). As no standardised experimental procedure exists for the determination of the Mode II fracture toughness, the ENF specimen dimensions and test protocol were adapted from the work of Yang et al. [35] and Gheibi et al. [26], (Figure 1b).

The initial crack length between the two MDPE substrates of *a* = 40 mm, was achieved using a 0.2 mm PTFE shim to prevent bonding [36]. Upon curing, the side of the lower substrate was scribed with vertical lines at 0.2 mm increments to create a scale for measuring crack length propagation during testing [35]. For the DCB test, the adherend section of the joint was unsupported during testing and the load, *P*, was applied to the specimen using two forks connected to pins inserted into predrilled holes in the tips of the substrates [26,29]. For the ENF test, the joints were placed on two supporting cylinders at a span of 140 mm and the load, *P*, was applied by a third cylinder at the mid-point of the specimen [35]. The joints were then loaded under a displacement control condition at a rate of 2.0 mm/min [37] and the crack length, *a*, was monitored using a 10× magnification USB digital microscope camera (Dino-Lite, Torrance, CA, USA) [36].

Following testing, the DCB and ENF results were used to determine the values of energy release rate and cohesive strength of the adhesive in tension (*G_n_*, *t_n_*) and shear (*G_s_*, *t_s_*) using the following equations [29,34,38,39]:

(1) Gn= Pn2EnInbn (3EnInδn2Pn)23.

(2) $$G_{s}=\;\frac{3P_{s}^{2}a_{s}^{2}}{2E_{s}b_{s}^{2}H_{s}^{3}}+\;\frac{3P_{s}v}{8b_{s}H_{s}}$$

(3) tn=δn−13  2Pn23EnInbn (3EnIndδndt2Pn)23

(4) $$t_{s}=\;\frac{dG_{s}}{d\delta_{s}}$$

where subscripts *n* and *s* indicate variables associated with peel (DCB) and shear (ENF), respectively. The geometry of the specimens is given by the height of the adherends, *H*, the width, *b*, and the crack length, *a*. *P* is the applied force, *ν* is the shear deformation of the adhesive layer and *E* and *I* are the Young’s modulus and the second moment of area of the adherend respectively. Finally, *δ_n_* is the displacement of the loading points for the DCB and *δ_s_* is the shear displacement of the adherends at the crack tip.

### 2.2. Tensile Lap-Shear Testing

**Figure 2:**
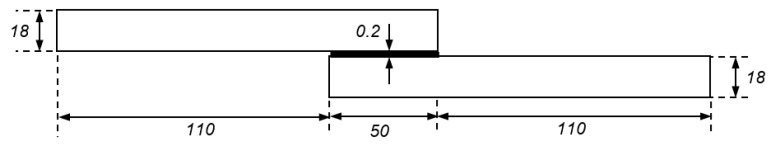
Specimen geometry for single lap-shear adhesive joint (dimensions in mm). Specimen breadth, *b* = 25 mm.

Tensile lap-shear joint specimens were prepared using the method described in Section 2.1 and in accordance with the ASTM D1002-99 standard using strips of MDPE pipe (160 mm long × 25 mm wide × 18 mm thick). The adherend strips were then bonded in a single lap-shear joint geometry with a nominal adhesion area of 25 × 50 mm, see Figure 2. Again, all tests were carried out at a crosshead speed of 2.0 mm/min and the resulting lap-shear strength in Pascals (*Pa*) was calculated as the measured peak load divided by the true surface area of the bond.

## 3. Numerical Analysis 

### 3.1. Analysis Conditions

**Figure 3:**
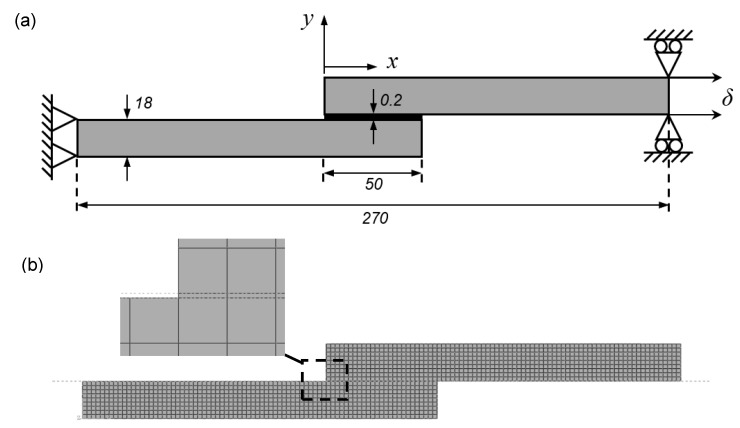
(**a**) Geometry and boundary conditions and (**b**) mesh detail for the single lap-shear adhesive joint (dimensions in mm).

The numerical analysis was carried out using the commercial FEM software ABAQUS^®^ (v2017) (Dassault Systèmes, Paris, France). The FEM model was developed using a CZM with triangular shape TSL to predict the strength of MDPE gas pipeline material adhesively bonded in a single lap joint configuration and to make a direct comparison with the results of the physical experiments detailed in Section 2.2. The numerical analysis was performed using non-linear geometrical considerations [40,41], with adherend and adhesive properties taken from the results of the characterisation tests detailed in Section 2.2. The geometry, mesh details and boundary conditions of the single lap joint model are depicted in Figure 3. The joint was simulated with a three-dimensional FE model, using eight-node reduced integration linear brick (C3D8R) elements for the MDPE adherends and a single row of eight-node three-dimensional cohesive elements (COH3D8) for the adhesive with an element thickness of 0.2 mm.

Prior to analysis of the lap-shear joint model, a mesh dependency study was undertaken to evaluate the influence of mesh refinement of the cohesive elements in the adhesive layer and ensure convergence of the solution [25]. The effect of increasing element lengths of 0.2–2.6 mm on the resulting maximum load (*P_m_*) was considered and the maximum deviation relative to the average (*P_m_/P_m avg_*) was 0.36%. This apparent mesh independence is typical for CZM modelling since an energetic criterion, based on the fracture toughness of the material (*G^c^*), is used for the damage growth [42]. As the energy required for crack propagation is averaged over the damaged area (rather than using a discrete value of maximum stress/strain), the result will be mesh independent provided that a minimum refinement is used [30,31]. Based on this convergence study it was determined that the appropriate size for the cohesive elements was 0.2 mm, thus, the final FEM model consisted of 25 cohesive elements and 1440 3D elements.

The boundary conditions were defined to represent the actual physical tensile test conditions as closely as possible. The joint was clamped at one edge, while the opposite edge was subjected to a tensile displacement with lateral restraining [43].

### 3.2. Cohesive Zone Model

**Figure 4:**
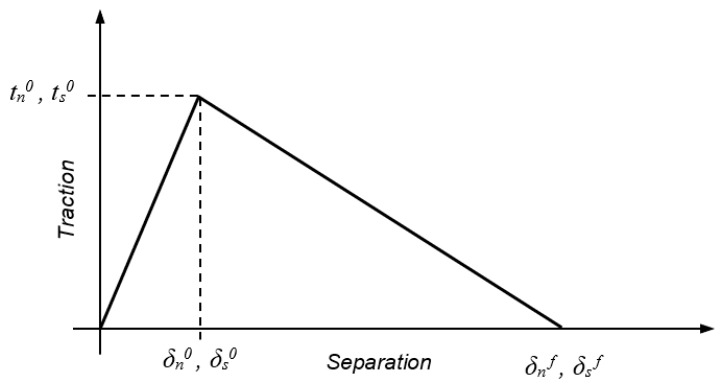
Triangular TSL with linear softening availble in ABAQUS^®^.

CZMs are used to simulate elastic loading, damage initiation and crack growth due local failure within a material [25]. They are based on the relationship between stresses and relative displacements connecting paired nodes of adhesive elements, to model elastic behaviour up to peak strength and subsequent softening of the material to failure [44]. In this work, a triangular shaped TSL was used to define this relationship between stresses and relative displacements of the CZM, see Figure 4, and assumes an initial linear behaviour up to the maximum cohesive strength in tension (*t_n_*^0^) or in shear (*t_s_*^0^) at initial displacements (*δ_n_*^0^, *δ_s_*^0^), followed by linear degradation to final displacements (*δ_n_^f^*, *δ_s_^f^*). The areas under the TSLs in tension and shear are equal to fracture toughness in tension (*G_n_^c^*) and in shear (*G_s_^c^*), respectively. 

The initial linear elastic behaviour of the TSL is defined by an elastic constitutive matrix (**K**), which contains the stiffness parameters and relates the stresses and strains in tension and shear across the interface [30,45]:

(5) $$t=\;\left\{\begin{array}{c} t_{n}\\ t_{s} \end{array}\right\}=\;\left[\begin{array}{cc} K_{nn} & K_{ns}\\ K_{ns} & K_{ss} \end{array}\right]\;.\;\left\{\begin{array}{c} \varepsilon_{n}\\ \varepsilon_{s} \end{array}\right\}=K\varepsilon$$

where *t_n_* and *t_s_* are the tensile and shear cohesive tractions and *ε_n_* and *ε_s_* are the tensile and shear strain. A suitable approximation for thin adhesive layers is provided with *K_nn_* = *E*, *K_ss_* = *G* and *K_ns_* = 0.

Damage initiation can be specified by different criteria [45], such as maximum nominal strain (MAXE), maximum principal strain (MAXPE), maximum principal stress (MAXPS), maximum nominal stress (MAXS), quadratic nominal strain (QUADE) and quadratic nominal stress (QUADS). In this study, the maximum nominal stress (MAXS) criteria was selected. The damage is assumed to initiate when the maximum nominal stress ratio reaches a value of one and can be represented as [45]:

(6) $$max\;\left\{\frac{t_{n}}{t_{n}^{0}},\frac{t_{s}}{t_{s}^{0}}\right\}=\;1$$

The Macaulay brackets are used to signify that a pure compressive deformation or stress state does not initiate damage [46].

Once peak cohesive strength has been attained and the criterion of equation (6) is met, the material stiffness is degraded. Damage propagation to complete separation is predicted using a linear power law form of the required energies for failure in the pure modes [45] and is represented as:

(7) $$\frac{G_{n}}{G_{n}^{c}}+\frac{G_{s}}{G_{s}^{c}}=\;1$$

When equation (7) is satisfied, stresses are completely released in the cohesive zone and, thus, new traction free crack faces are generated [26].

## 4. Results and Discussion

### 4.1. Materials Characterisation

**Figure 5:**
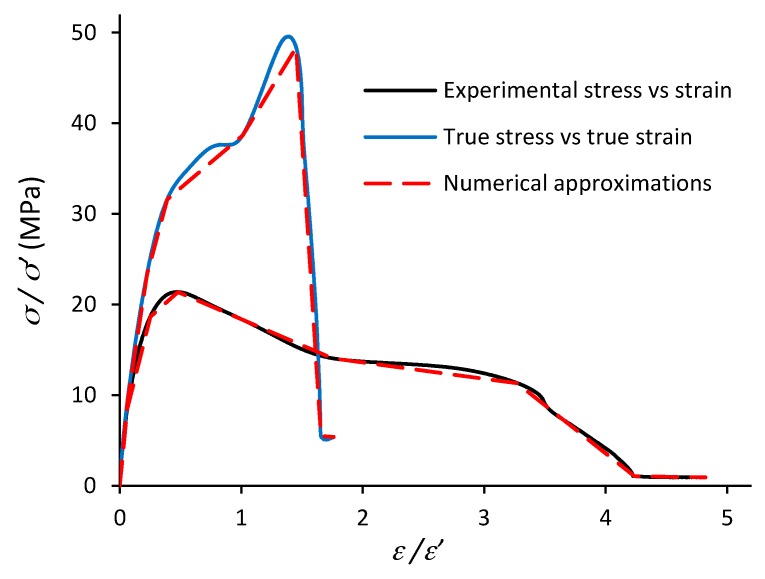
Stress-strain curves for the MDPE adherend and numerical approximation for the FEM analysis.

The bulk stress-strain (*σ–ε*) response of the MDPE adherends obtained via tensile testing of five specimens is presented in Figure 5. The average tensile strength was 21.4 MPa, which is within the range specified by the supplier in Table 1, and the Young’s modulus was calculated as 700 MPa. Figure 5 also shows the calculated True stress-strain (*σ’–ε’*) curve for the MDPE, along with the linear numerical approximation that was used as an input to the FEM model to simulate classical Mises plasticity.

**Figure 6:**
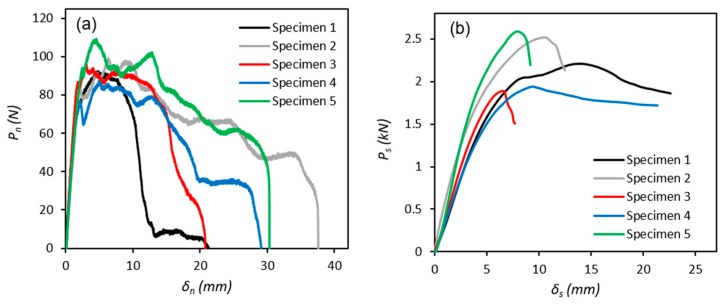
Load-displacement (*P–δ*) responses for five specimens each of (**a**) DCB and (**b**) ENF tests.

Experimental load-displacement (*P–δ*) responses of five valid DCB and ENF specimens, which all exhibited cohesive failure are presented in Figure 6. The results are of typical shape for mode I and II fracture [26] with peak loads in the range 87.3–109 N and 1.88–2.58 kN respectively. The results of the DCB tests display a wider post-peak range than those of the ENF tests due to the inconsistent peel test performance of the PE:PE adhesive joints.

**Figure 7:**
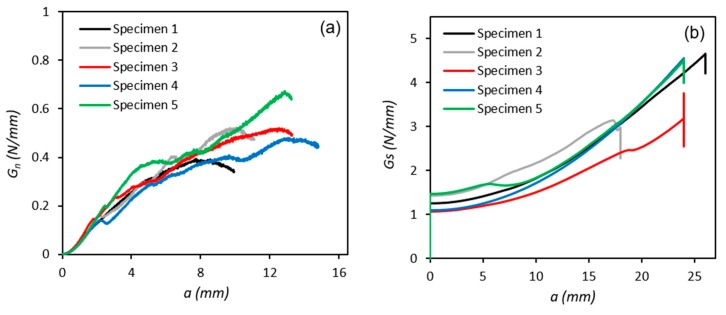
Fracture energy curves for (**a**) DCB and (**b**) ENF tests.

**Figure 8:**
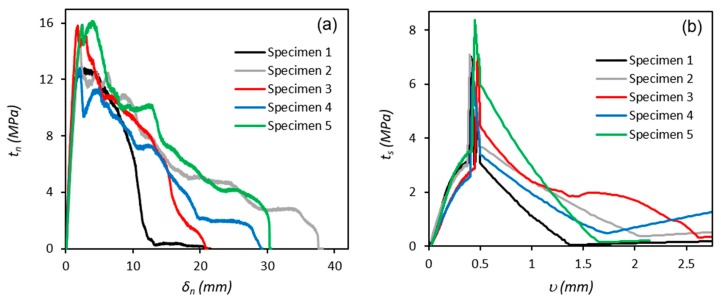
Cohesive strength curves for (**a**) DCB and (**b**) ENF tests.

Following testing, Mode I and II fracture energy release rates and the cohesive strength of the adhesive in tension (*G_n_*, *t_n_*) and shear (*G_s_*, *t_s_*) were calculated using Equations (1)–(4), (Figure 7 and Figure 8). In general, for the DCB tests, the fracture energy increases with increasing crack length until it reaches its maximum value (*G_n_^c^*) or steady state toughness, where the cohesive stresses become zero [47]. For the ENF tests, the fracture energy increases exponentially with increasing crack length until it reaches a maximum value, at which point unstable crack propagation takes place, manifested in a sudden drop in fracture energy [48].

The critical toughness (*G^c^*) of the adhesive in modes I and II were extracted from Figure 7a,b respectively. The maximum cohesive strength (*t*^0^) of the adhesive in modes I and II were extracted from Figure 8a,b respectively. The values of these parameters obtained for each test in mode I and II are summarised in Table 2 and the calculated average values are used to specify the corresponding triangular shape TSLs for input into the FE model in Section 4.3.

### 4.2. Tensile Lap-Shear Tests

**Figure 9:**
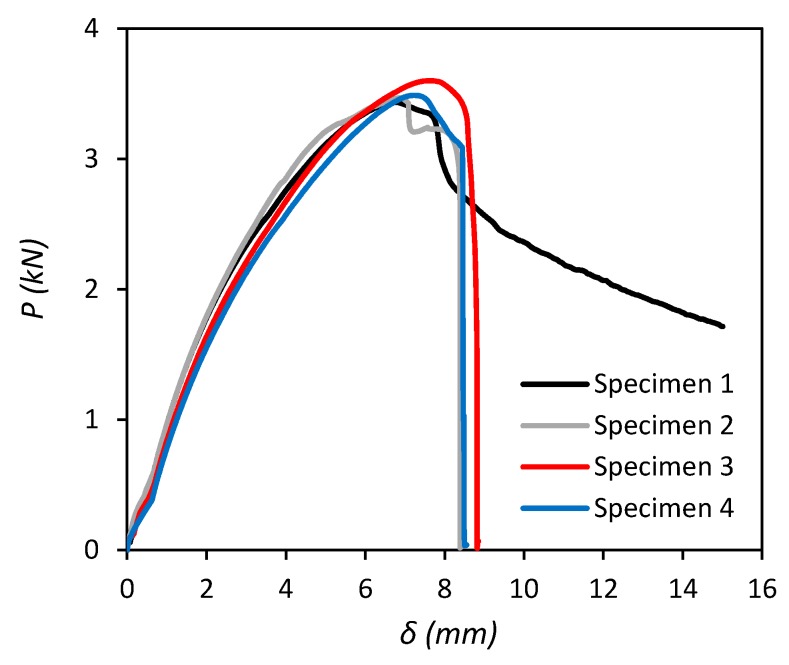
Load displacement responses for tensile lap-shear tests.

Load-displacement (*P–δ*) responses of four tensile lap-shear tests are presented in Figure 9. The maximum load was in the range of 3.46–3.60 kN for the four test specimens and the average load and standard deviation for the sample were 3.497 kN and 0.072 kN, respectively. Three specimens failed in a similar nature with a maximum displacement of 7–8 mm in the MDPE substrate up to peak load before the adhesive underwent brittle failure. For specimen #1, the MDPE substrates continued to displace up to 15 mm after reaching peak load and the adhesive joint did not fully separate. The results of these four test specimens are used to validate the results of the FE model in Section 4.3.

### 4.3. Numerical Model

Table 3 summarises the cohesive properties of the acrylic adhesive established from the experimental results of the DCB and ENF tests in Section 4.1. These values specify the mode I and II TSLs for input into the proceeding FEM simulations and strength predictions. Since it is known that the cohesive properties of adhesives are highly dependent on thickness (*h*) [44], It should be mentioned that the parameters reported in Table 3 are only valid for a value of *h* = 0.2 mm. It should be noted that since the tensile lap-shear test is dominated by mode II failure, the restriction of lateral contraction [49] was not considered important in this study and the stiffness of the CZM was taken as the elastic modulus of the adhesive, E.

**Figure 10:**
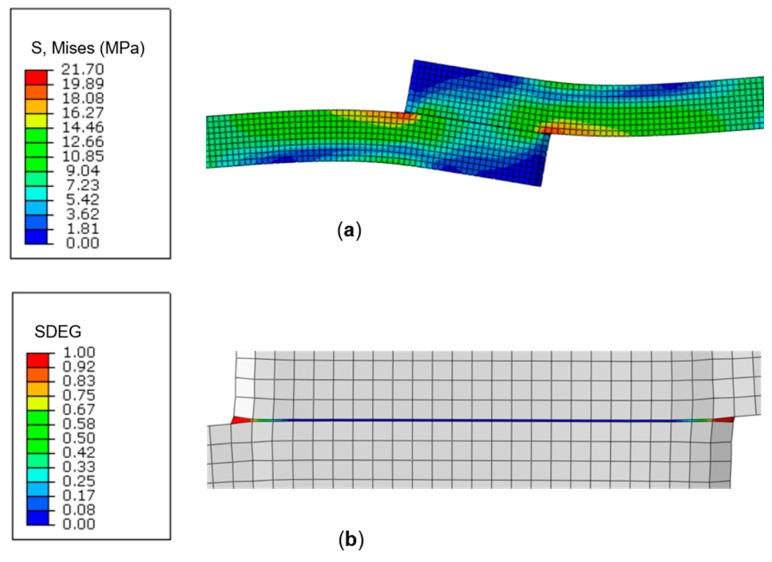
(**a**) Von Mises stress and (**b**) damage variable distribution (SDEG) contour plots for the lap-shear joint model.

Contour plots of von Mises stress and damage (SDEG) distribution for the lap-shear joint model are presented in Figure 10a,b, respectively. The plots reveal that maximum stress occurs at the joint overlap edges, forming a concentration that initiates damage, leading to cohesive crack propagation and fracture in the adhesive bond [26].

**Figure 11:**
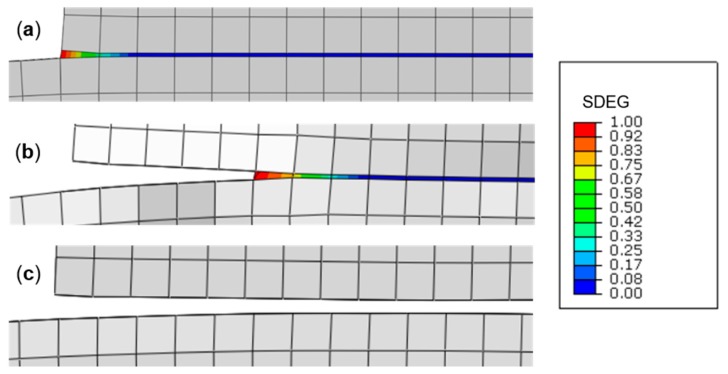
Progressive failure process of the adhesive layer in the lap-shear joint, (**a**) damage initiation at the overlap edges (SDEG-% = 0), (**b**) propagation towards the joint centre (SDEG-% ≈ 40), (**c**) joint failure (SDEG-% = 100).

The damage variable, SDEG, in Figure 10b corresponds to the stiffness degradation in the adhesive layer, and ranges from SDEG = 0 for the undamaged material, i.e., the initial elastic portion of the traction separation law, to SDEG = 1 for complete failure, i.e., cohesive stress is zero and the separation displacement is at the maximum. The progressive failure process of the adhesive layer in the lap-shear joint (SDEG-% = 0 to 100) is illustrated in Figure 11 (only one half of the joint is shown for clarity). It is clearly shown that damage is initiated cohesively in the adhesive bond at the overlap edges (Figure 11a) and propagates towards the centre of the bond (Figure 11b) leading to complete failure of the joint (Figure 11c).

**Figure 12:**
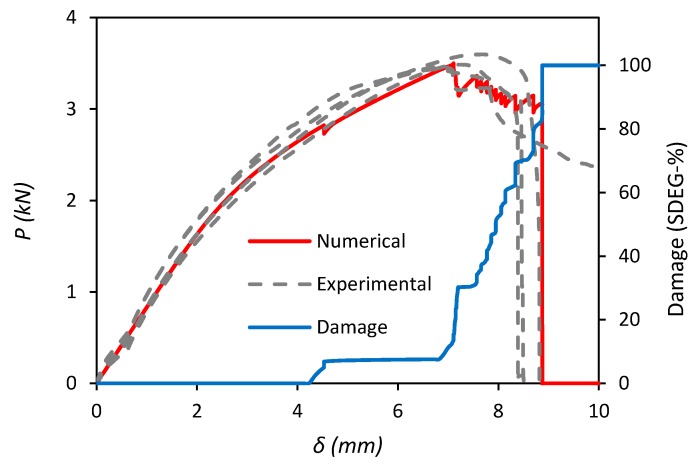
Load-displacement (*P–δ*) responses of the lap-shear joint for numerical simulation, damage evolution (SDEG-%) and experimental tests.

The numerical load-displacement (*P–δ*) response of the lap-shear joint, together with the progressive damage evolution of the adhesive bond (SDEG-%) are presented in Figure 12. The result shows an initial region of elastic strain in the MDPE adherends before the onset of progressive plastic deformation at *δ* = 7 mm. The first sign of damage initiation (SDEG-% = 10) in the adhesive bond occurs at a displacement of 4.5 mm under a load of 2.8 kN and then remains stable as the MDPE adherends continue to deform up to maximum load, *P_m_* = 3.5 kN at *δ* = 7 mm. At this point the adhesive bond begins to fail rapidly, with a sudden 20% increase in overall damage, quickly followed by progressive failure of the cohesive elements to 100% damage and ultimate failure of the joint at *δ* = 8.7 mm.

A comparison between the load-displacement (*P–δ*) responses of the numerical simulation and experimental tests of the lap-shear joint is also presented in Figure 12. It can be seen that the FE model is able to accurately predict the physical test results for the adhesive joint in terms of capturing all of the relevant features of the failure process, such as the stiffness of the adherends, the maximum load (*P_m_*) sustained by the specimens and the failure displacement. The percentile differences between the numerical and the four experimental results for *P_m_* are reasonably small in the range from −2.89% to +1.84%.

## 5. Conclusions

The main objective of this work was to develop an FE model that can accurately predict the strength of MDPE gas pipeline material adhesively bonded in a single lap joint configuration using a MMA based structural adhesive.

Physical experiments were undertaken to determine important properties of the MDPE and adhesive materials for input into the FE model. Firstly, the bulk stress-strain (*σ–ε*) response of the MDPE adherend was obtained via tensile testing to provide a linear numerical approximation to simulate the plastic deformation of the material. The average tensile strength of the MDPE was 21.4 MPa and the Young’s modulus was 700 MPa. Next, the energy release rate and cohesive strength of the adhesive in failure modes I and II were directly extracted utilising the mechanical responses of DCB and ENF fracture tests. The critical toughness (*G^c^*) and maximum cohesive strength (*t*^0^) were in the range 0.39–0.67 N/mm and 12.83–16.16 MPa in Mode I (DCB) and 3.14–4.65 N/mm and 8.45–10.07 MPa in Mode II (ENF). The averages of these values were then used to specify the corresponding triangular shape TSLs for input into the FE model.

The proposed model was implemented in ABAQUS^®^ using a CZM with a triangular shape TSL and numerically it predicted the mechanical response of the single lap-shear test specimens with high accuracy in terms of the stiffness of the adherends, the maximum load and the failure displacement. The maximum load predicted by the model was 3.499 kN and the average value for the four test specimens was 3.497 kN, with maximum difference for individual specimens of <3%. 

Overall, it can be concluded that the FE model developed in this work is suitable for modelling progressive damage simulation and predicting the strength of adhesively bonded MDPE gas pipeline material in a single lap-shear joint configuration. However, the single lap-shear configuration is dominated by mode II failure and although this is representative of a typical gas pipeline joint and is the reason it was selected for this investigation, it should be noted that the entire CZM cannot be considered fully validated without also considering mode I failure.

The results of this study will form the foundation for future work to develop the numerical model for the simulation of full-scale adhesively-bonded MDPE gas pipeline joints and tapping tees for direct comparison with traditional electrofusion welded parts. This future work will also consider the effects different loading conditions such as torsion and impact, environmental conditions, such as temperature and humidity.

## Figures and Tables

**Table 1:** Key properties of the MDPE substrate and acrylic adhesive (Data from [11]).

**MDPE Substrate (PE80 Yellow Gas Pipeline)**	
Dimensions	250 mm diameter, 18 mm thick
Density	0.93–0.95 g/cm^3^
Molecular Weight	16 × 10^4^
Polydispersity index	16
Melt flow rate (190 °C/5 kg)	1.0 g/10 min
Tensile strength	14–22.8 MPa
**Acrylic Adhesive (Weicon Easy-Mix PE-PP 45)**	
Mixing ratio by volume	10:1 (resin/hardener)
Density	1.07 g/cm^3^
Viscosity at +20 °C	45 mPa.s
Pot life (10 mL at +20 °C)	2–3 min
Glass transistion temperature (Tg)	35 °C
Processing temperature (optimal)	+20 to +25 °C
Curing Temperature	+15 to +70 °C
Curing time at 20 °C (for PP substrates)	2–3 h—handling strength (35% of final)
	6 h—mechanical strength (50% of final)
	24 h—final strength (100% cured)

**Table 2:** Mode I and II cohesive parameters extracted from DCB and ENF tests.

Specimen No.	Mode I (DCB)		Mode II (ENF)	
	*G_n_^c^* (N/mm)	*t_n_*^0^ (MPa)	*G_s_^c^* (N/mm)	*t_s_*^0^ (MPa)
1	0.39	12.83	4.65	10.02
2	0.52	14.67	3.14	9.47
3	0.52	15.84	3.76	9.50
4	0.48	12.83	4.56	8.45
5	0.67	16.16	4.53	10.07
Average	0.52	14.46	4.13	9.50
Std Dev.	0.10	1.60	0.66	0.65

**Table 3:** Properties of the Weicon PE–PP 45 adhesive used for CZM modelling.

Property	Weicon PE-PP 45
E (MPa)	1850
G (MPa)	560
*t_n_*^0^ (MPa)	14.46
*t_s_*^0^ (MPa)	9.50
*G_n_*^0^ (N/mm)	0.52
*G_s_*^0^ (N/mm)	4.13
